# Antibacterial and Cytocompatible: Combining Silver Nitrate with Strontium Acetate Increases the Therapeutic Window

**DOI:** 10.3390/ijms23158058

**Published:** 2022-07-22

**Authors:** Marjan Kheirmand Parizi, Katharina Doll, Muhammad Imran Rahim, Carina Mikolai, Andreas Winkel, Meike Stiesch

**Affiliations:** 1 Department of Prosthetic Dentistry and Biomedical Materials Science, Hannover Medical School, Carl-Neuberg-Strasse 1, 30625 Hannover, Germany; doll.katharina@mh-hannover.de (K.D.); rahim.muhammad@mh-hannover.de (M.I.R.); mikolai.carina@mh-hannover.de (C.M.); winkel.andreas@mh-hannover.de (A.W.); 2 Lower Saxony Center for Biomedical Engineering, Implant Research and Development (NIFE), Stadtfelddamm 34, 30625 Hannover, Germany

**Keywords:** therapeutic window, silver, strontium, peri-implantitis, osseointegration, antibacterial, cytocompatible

## Abstract

Microbial infection and insufficient tissue formation are considered to be the two main causes of dental implant failure. Novel studies have focused on designing dual-functional strategies to promote antibacterial properties and improve tissue cell response simultaneously. In this study, we investigated the antibacterial properties and cytocompatibility of silver nitrate (AgNO_3_) and strontium acetate (SrAc) in a mono-culture setup for dental application. Additionally, we defined the therapeutic window between the minimum inhibitory concentration against pathogenic bacteria and maximum cytocompatible dose in the case of combined applications in a co-culture setup. Antibacterial properties were screened using *Aggregatibacter actinomycetemcomitans* and cell response experiments were performed with osteoblastic cells (MC3T3) and fibroblastic cells (NIH3T3). The osteoinductive behavior was investigated separately on MC3T3 cells using alizarin red staining. A therapeutic window for AgNO_3_ as well as SrAc applications could be defined in the case of MC3T3 cells while the cytocompatibility of NIH3T3 cells was compromised for all concentrations with an antibacterial effect. However, the combined application of AgNO_3_/SrAc caused an enhanced antibacterial effect and opened a therapeutic window for both cell lines. Enhanced mineralization rates could be observed in cultures containing SrAc. In conclusion, we were able to demonstrate that adding SrAc to AgNO_3_ not only intensifies antibacterial properties but also exhibits bone inductive characteristics, thereby offering a promising strategy to combat peri-implantitis and at the same time improve osseointegration in implant therapy.

## 1. Introduction

Nowadays, dental implants represent a common treatment to replace missing teeth. However, certain complications and challenges are associated with this treatment option. The median prevalence of peri-implantitis, as the most frequent complication reported in implant dentistry, ranged from 7% among healthy populations to 38.4% in subjects with 10 year functional implants [1]. More severe risk factors such as smoking, systemic diseases, and poor oral hygiene can negatively impact the peri-implantitis prevalence rate [1]. Inflammatory processes caused by bacterial colonization damage both hard and soft tissues at the implant’s site and have been described as a major contributor to low success rates [2]. In addition, the success rate of dental implants further relies on sufficient initial osseointegration that determines prolonged stability [3]. As a consequence, optimized tissue integration as well as limited bacterial colonization are considered key factors for a sustainable functional implant [4,5]. In an effort to reduce dental implant failure, mutual implant functionalization strategies should be considered which address anti-bacterial properties as well as tissue regeneration at the same time.

Silver, as one of the most effective antimicrobial metals, has been extensively employed in implant research due to its broad-spectrum activity and long-term stable antibacterial ability [6]. Accordingly, until the year 2016, 37.8% of studies used different forms of silver in antimicrobial dental implant functionalization strategies [7]. On the other hand, several investigations have reported the dose-dependent toxic effect of silver not only on bacteria but also on tissue cells [8,9]. The minimal inhibitory concentration (MIC) of silver towards procaryotic cells usually overlaps with the acceptable biocompatible concentration for eucaryotic cells, which limits any potential therapeutic window to a narrow range [10]. Currently, two different approaches are used in order to increase the therapeutic window for silver administration: (i) changing the physicochemical properties of the applied silver (e.g., shape, size, electrochemistry, and concentration) or (ii) incorporating a secondary element or bioactive chemical to reduce cell toxicity of silver while enhancing tissue formation but maintaining antibacterial properties [11]. Organic bioactive chemicals (e.g., hydroxyapatite (HA), chitosan (CS)), inorganic elements (e.g., zinc (Zn), copper (Cu), gold (Au), and strontium (Sr)), or various combinations of them have been incorporated with different chemical forms of silver in order to reach the optimal balance between antibacterial behavior, chemical biocompatibility, and osteogenic response [12,13,14,15,16,17,18,19,20]. Among these elements, strontium as an alkaline metal with a strong inductive effect on bone tissue appears to be a particularly promising agent. It not only concurrently impedes bone resorption by suppressing osteoclast activity but can also enhance bone formation by increasing osteoblast function [21]. Several studies have already demonstrated the bone formative properties of strontium-leaching biomaterials in clinical settings [22]. Additionally, some in vitro investigations also reported an antibacterial impact of strontium; amongst them, one recent investigation was carried out on bacteria associated with dental implant infections [23,24,25,26].

The combination of silver and strontium was previously assessed in some studies for antibacterial activity and cytocompatibility when applied as surface coating [16,20,24,27,28,29]. Although the majority of these investigations reported good antibacterial effects and biocompatibility, their application of coated surfaces did not include the definition of the optimum concentrations of both metals in order to determine the range of a desired therapeutic window. The biological effects or interactions of these two chemicals (regardless of coating effects in case of chemical composition or surface topography) separately and in combination were not assessed in a comprehensive manner. Furthermore, the antibacterial activity of combined silver/strontium against oral pathogens related to dental peri-implantitis and, thus, the specific therapeutic window for this application in dentistry has not been investigated so far.

Therefore, the present study aimed to systematically analyze both the antibacterial effect and the cytocompatibility of silver, strontium, as well as combinations of both within one study to define the therapeutic window in mono-culture and co-culture setups for dental application. The antibacterial activity was evaluated against *Aggregatibacter actinomycetemcomitans*, one of the major bacterial pathogens in dental peri-implantitis [30]. At the same time, the cellular cytotoxicity of the different metal combinations was determined for fibroblasts and osteoblasts, which are the most relevant cells for dental implant studies [31]. Finally, the most promising concentrations were analyzed in a co-culture assay containing both bacterial and tissue cells, and osteogenic differentiation was also addressed.

## 2. Results

### 2.1. Antibacterial Activity and Cytocompatibility of Silver Nitrate

**Figure 1:**
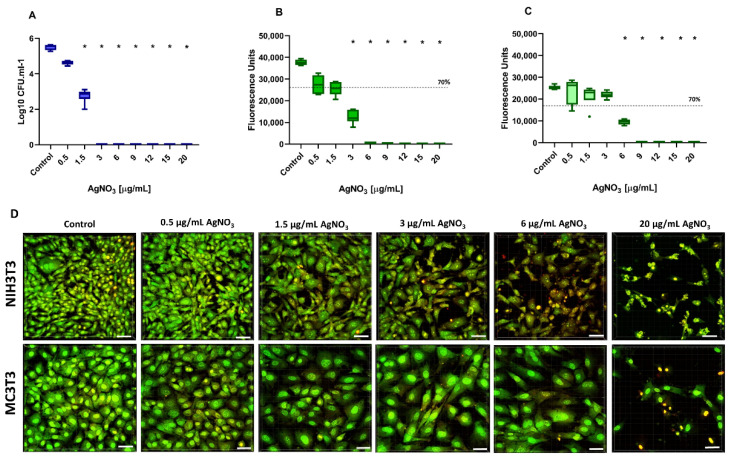
Antibacterial Activity and Cytocompatibility of Silver Nitrate. Tukey Box Plots of (**A**) bacterial colony numbers (expressed as log CFU/mL) of *A. actinomycetemcomitans* and cell metabolic activity determined by CellTiter-Blue assay of (**B**) NIH3T3 and (**C**) MC3T3 after 24 h incubation with AgNO_3_ at different concentrations. The dashed lines represent 70% of cell viability. Black stars indicate statistically significant decreases compared to the control with *p* ≤ 0.05. (**D**) BacLight staining of NIH3T3 and MC3T3 cells treated with corresponding AgNO_3_ concentrations. The samples were examined under the CLSM. Scale bars: 50 μm.

In this study, the effects of different combinations of additives were tested regarding antibacterial properties and cytotoxicity in mono-culture and co-culture settings to define putative therapeutic windows. In order to ensure the best possible comparability of the results, all experiments were performed in the same mixed culture medium (DMEM + αMEM, 1:1), which was selected in preliminary experiments. The antibacterial effect of AgNO_3_ at concentrations of 0.5, 1.5, 3, 6, 9, 12, 15, and 20 µg/mL against the periodontal pathogen *A. actinomycetemcomitans* was evaluated after a 24 h incubation period by counting the number of bacterial colonies (CFU) grown on FAA plates (Figure 1A). A dose-dependent antimicrobial effect on *A. actinomycetemcomitans* was determined. Treatment with AgNO_3_ concentrations of 0.5 µg/mL resulted in a reduction of 87% in bacterial colony numbers, but did not reach statistical significance (*p* = 0.051). With an increasing concentration of AgNO_3_ (≥1.5 µg/mL), a statistically significant reduction was observed. No viable bacteria could be detected at AgNO_3_ concentrations of ≥3 µg/mL, indicated by 100% growth inhibition. Therefore, the minimum inhibitory concentration (MIC), which we defined as the lowest concentration with statistically significant CFU reduction, of AgNO_3_ in this mono-culture assay was set as 1.5 µg/mL.

Parallel to the antibacterial effect, the cellular viability of fibroblasts (NIH3T3) and osteoblasts (MC3T3) following exposure to AgNO_3_ was investigated at the same concentration range using the CTB viability assay (Figure 1B,C). Statistically significant viability reduction was detected for AgNO_3_ concentrations of ≥3 µg/mL in the case of NIH3T3 cells and for concentrations of ≥6 µg/mL in case of MC3T3 cells. However, a reduction of more than 30% of cell viability was already considered as a cytotoxic effect according to DIN ISO 10993-5 [32]. In this regard, an AgNO_3_ concentration of ≥1.5 µg/mL (for NIH3T3) and ≥6 µg/mL (for MC3T3) must be considered cytotoxic. To support these observations, cell fluorescence staining and CLSM microscopy were used to document morphological alterations following exposure to test substances (Figure 1D). The microscopy results confirmed cell compatibility at low concentrations of AgNO_3_ (0.5 µg/mL) in both cell lines with normal elongated morphologies and green fluorescence, but different sensitivities when using higher concentrations. In the case of NIH3T3, AgNO_3_ concentrations ≥1.5 µg/mL led to reduced cell numbers, rounded cell structures, and a slightly reddish color, which became even more intense as the dose increased (Figure 1D). Such visible morphological changes could not be observed for MC3T3 up to a concentration of 6 µg/mL. According to the combined results of antibacterial and cytotoxicity testing, a desired therapeutic window, defined as the concentration interval between MIC and the maximum cytocompatible dose of AgNO_3_, could be determined as from ≥1.5 µg/mL to <6 µg/mL for MC3T3 cells. No therapeutic window could be identified for NIH3T3 cells in this mono-culture setup.

### 2.2. Antibacterial Activity and Cytocompatibility of Strontium Acetate

**Figure 2:**
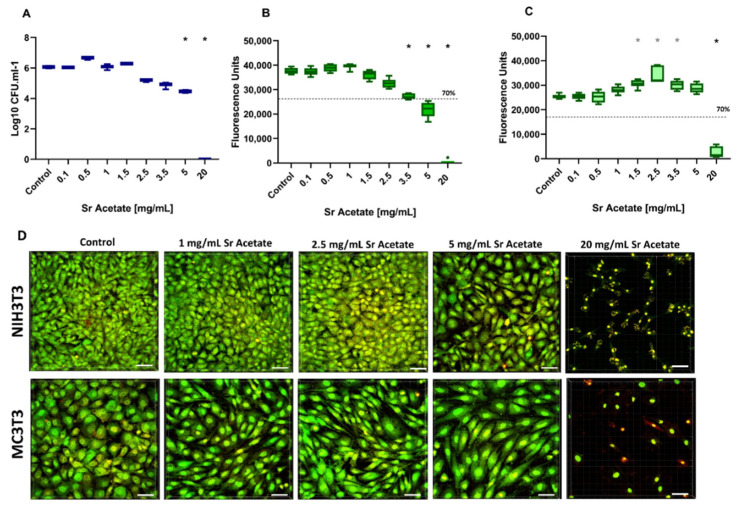
Antibacterial Activity and Cytocompatibility of Strontium Acetate. Tukey Box Plots of (**A**) *A. actinomycetemcomitans* growth given as log CFU/mL measured after 24 h inoculation. Metabolic activity of (**B**) NIH3T3 and (**C**) MC3T3 cell lines cultivated in the presence of Sr Acetate with different concentrations. The dashed lines represent 70% of cell viability. Black and grey stars indicate statistically significant decreases and increases, respectively, compared to the control with *p* ≤ 0.05. (**D**) BacLight staining of NIH3T3 and MC3T3 treated with corresponding Sr Acetate concentrations. The samples were examined under the CLSM. Scale bars: 50 μm.

Similar to the analyses for AgNO_3_, the effects of SrAc on bacteria and tissue cells were initially tested in mono-culture approaches. For *A. actinomycetemcomitans*, a dose-dependent decrease in CFU/mL could be observed within the range of 0.1 to 20 mg/mL. This reduction became statistically significant at SrAc concentrations ≥5 mg/mL (Figure 2A). Thus, SrAc showed an MIC of 5 mg/mL in the mono-culture experiment.

Cellular metabolic activity was again quantified using CTB assay. Regarding cytocompatibility, it could be observed that the viability of NIH3T3 cells did not diminish in comparison to non-treated cells up to SrAc concentrations ≤3.5 mg/mL. A reduction of more than 30% in metabolic activity was detected following treatments with concentrations ≥5 mg/mL (Figure 2B). The metabolic activity of MC3T3 cells initially significantly increased with increasing concentrations of SrAc (1.5, 2.5, and 3.5 mg/mL) compared to the control group (*p* = 0.003, *p* = 0.0002, and *p* = 0.008, respectively). Additionally, in contrast to fibroblasts, a significant reduction in viability was only detected at 20 mg/mL which exceeded 30%. The qualitative analysis using CLSM images of stained cells confirmed the results of the quantitative CTB assay (Figure 2D). At up to 2.5 mg/mL, no dominant alterations in morphology or visible cell amounts could be observed neither for NIH3T3 nor MC3T3. Beginning with 5 mg/mL, the fibroblasts appeared slightly rounded and fewer in number, whereas only isolated reddish dots remained in 20 mg/mL. In the case of MC3T3, a collapse of the cell culture was not indicated at lower concentrations and was observed exclusively for 20 mg/mL. However, it should be mentioned that the osteoblasts appeared slightly more elongated with aligned orientation when adding SrAc in a concentration range from 1 to 5 mg/mL (Figure 2D). Taken together, SrAc concentrations of ≤3.5 mg/mL (for NIH3T3) and ≤5 mg/mL (for MC3T3), respectively, were shown to be cytocompatible, indicating a possible therapeutic window of around 5 mg/mL in the case of MC3T3 cells. No therapeutic window could be defined following SrAc administration on NIH3T3.

### 2.3. Cell Viability in Combined Treatment with Silver Nitrate and Strontium Acetate

**Figure 3:**
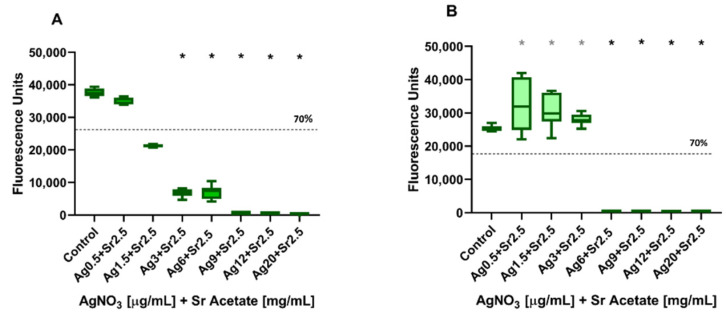
Cell viability of Silver Nitrate and Strontium Acetate in combination. CellTiter-Blue assay of (**A**) NIH3T3 and (**B**) MC3T3 treated with increasing concentrations of AgNO_3_ and 2.5 mg/mL Sr Acetate after 24 h. The dashed lines represent 70% cell viability. Black and grey stars indicate statistically significant decreases and increases, respectively, compared to the control with *p* ≤ 0.05.

The cytotoxic effects of a combined challenge with AgNO_3_ and SrAc were screened using NIH3T3 or MC3T3 cells before suitable concentrations were also tested for antibacterial effects in a co-culture approach, since cellular viability could not be addressed in a co-culture setup due to artefacts caused by bacterial viability. For this purpose, the cytocompatible concentration of SrAc for both cell lines (2.5 mg/mL) was combined with increasing concentrations of AgNO_3_ (0.5, 1.5, 3, 6, 9, 12, and 20 µg/mL), which was applied to the cells and analysed using CTB assay as well as CLSM microscopy as described before. Against the background stimulation of 2.5 mg/mL SrAc, the addition of AgNO_3_ ≥ 3 µg/mL resulted in a significant reduction in metabolic activity, while 0.5 µg/mL AgNO_3_ remained non-cytotoxic for NIH3T3 cells (Figure 3A). In the case of MC3T3 cells, AgNO_3_ concentrations ≥6 µg/mL appeared cytotoxic when combined with 2.5 mg/mL SrAc as indicated by the cell viability reduction of 30% (Figure 3B). Additionally, the previously observed significant increase in the metabolic activity of MC3T3 in the presence of 2.5 mg/mL SrAc could be confirmed and was not compromised by AgNO_3_ concentrations ≤3 µg/mL. When comparing the fluorescence units in Figure 1 and Figure 3 (effects of AgNO_3_ in different concentrations with and without adding SrAc 2.5 mg/mL) for NIH3T3, a significant increase in cell metabolism could be observed with 0.5 µg/mL AgNO_3_ in the presence of SrAc. The same effect could be detected for MC3T3 after treatment with AgNO_3_ at concentrations of 0.5 µg/mL, 1.5 µg/mL, and 3 µg/mL. In contrast, for cell cultures with higher concentrations of AgNO_3_ (NIH3T3: 3µg/mL AgNO_3_; MC3T3: 6 µg/mL AgNO_3_), metabolic activity was reduced when additionally stimulated with 2.5 mg/mL SrAc (Figure 1 and Figure 3).

### 2.4. Antibacterial Effect and Cytocompatibility of Combined Treatment with Silver Nitrate and Strontium Acetate in a Co-Culture System

**Figure 4:**
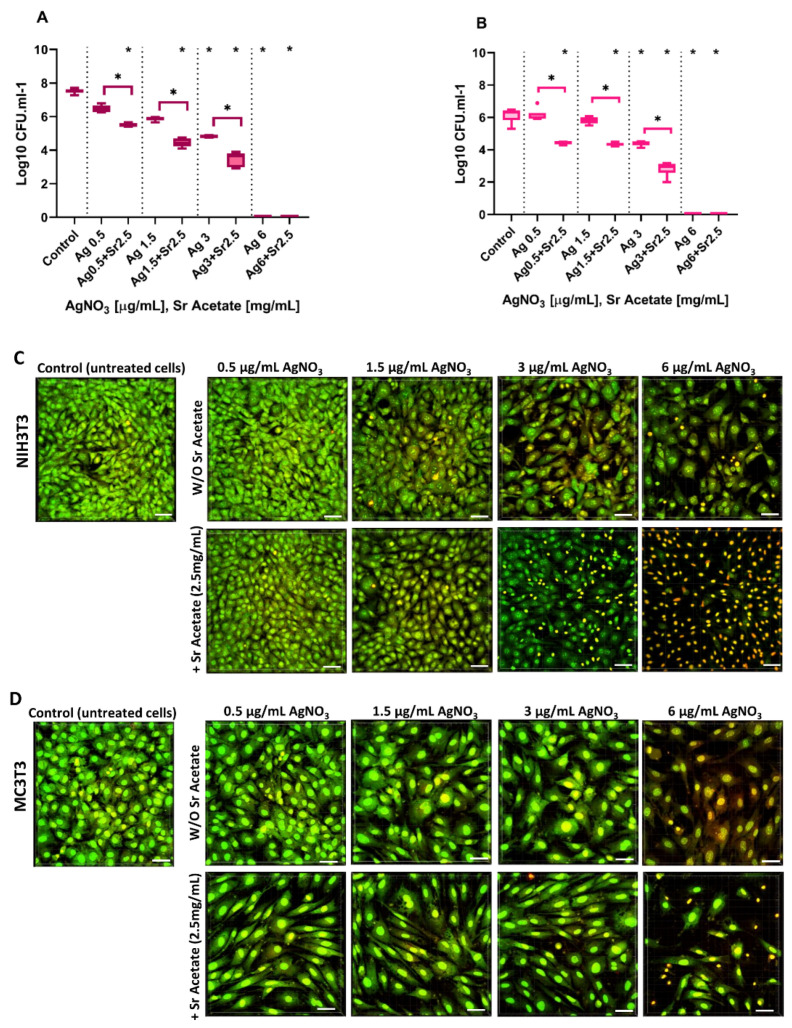
Antibacterial Effect and Cytocompatibility in Co-culture system. Bacterial growth in co-culture setup measured as log10 CFU/mL (**A**): NIH3T3, (**B**): MC3T3. Black stars show statistically significant decreases compared to the control with *p* ≤ 0.05. Brackets show statistically significant differences within groups with the same AgNO_3_ concentration but with or without Sr Acetate addition with *p* ≤ 0.05. NIH3T3 (**C**) or MC3T3 (**D**) were simultaneously co-cultured with *A. actinomycetemcomitans* and AgNO_3_ in two different groups (with or without adding 2.5 mg/mL Sr Acetate). Morphological alteration of co-cultured cells in different groups was imaged after 1 day by BacLight staining and CLSM. Scale bars: 50 μm.

Based on all results of the mono-culture experiments, for both cell lines, a co-culture setup with *A. actinomycetemcomitans* in the presence of 2.5 mg/mL SrAc was considered appropriate to investigate the effect of AgNO_3_ concentrations in the range of 0.5 to 6 µg/mL and to identify a possible therapeutic window for future applications. Similar to mono-culture experiments, the MIC regarding *A. actinomycetemcomitans* was measured by analyzing significant reductions in log CFU/mL (Figure 4A,B), and fluorescence staining followed by CLSM microscopy were used to determine cell morphology of both cell lines (Figure 4C,D). A statistically significant reduction in bacterial growth was observed in co-cultures with both cell lines after treatment with AgNO_3_ alone for concentrations ≥3 mg/mL (Figure 1). In contrast, bacterial growth was already statistically significantly reduced with a combined stimulus of 0.5 µg/mL AgNO_3_ together with 2.5 mg/mL SrAc. Therefore, this concentration could be defined as the MIC value for combination treatment. Such a decrease in bacterial growth in the presence of 2.5 mg/mL SrAc could be detected for any investigated AgNO_3_ treatment (Figure 4A,B). Regarding the cytocompatibility analysis, only CLSM imaging was performed (see above) and color change as well as morphological alterations were evaluated (Figure 4C,D). When NIH3T3 cells in the co-culture setup were treated with AgNO_3_ alone, only a concentration of 0.5 µg/mL caused no changes in cell shape and microscopically visible cell amounts. In the presence of SrAc, an AgNO_3_ concentration of 1.5 µg/mL still had no visible effect. However, if treated with AgNO_3_ ≥ 1.5 µg/mL (or ≥3 µg/mL in combination with 2.5 mg/mL SrAc), cells appeared more rounded and reddish in color. This decrease in viability was more pronounced when SrAc was added and confirmed its ambivalent effect regarding attenuation (with low AgNO_3_ concentrations) or enforcement (with high concentrations of AgNO_3_) of cytotoxicity observed in the context of silver treatment for both cell lines (Figure 4C,D). For MC3T3 cells in the co-culture setup, treatment with AgNO_3_ up to 3 µg/mL showed no changes in visible cell numbers independent of additional SrAc stimulation. As in the mono-culture, only the morphology of cells was affected by the addition of SrAc as cells appeared more elongated and aligned (Figure 4D). A visibly decreased amount of MC3T3 and more reddish stained cells were detected only when treated with 6 µg/mL AgNO_3_, an effect enhanced again in the presence of SrAc (similar to the findings in the mono-culture and confirming the results of the CTB assay). According to these data, cytocompatible doses of AgNO_3_ in combination with 2.5 mg/mL SrAc could be defined as <3 µg/mL for NIH3T3 and <6 µg/mL for MC3T3. For MC3T3, this concentration was in line with the CTB analysis of combined treatment in the mono-culture (Figure 3), whereas NIH3T3 appeared to be less affected in the co-culture setup. Regarding an inferable therapeutic window in the presence of 2.5 mg/mL SrAc, it was shifted for MC3T3 cells towards lower concentrations of AgNO_3_ (0.5–3 µg/mL) and for NIH3T3 cells it opened in a range of 0.5–1.5 µg/mL AgNO_3_. Thus, 0.5–1.5 µg/mL AgNO_3_/SrAc (2.5 mg/mL) could be identified as a common therapeutic window for both cell lines.

### 2.5. Osteogenic Differentiation of MC3T3 Cells

**Figure 5:**
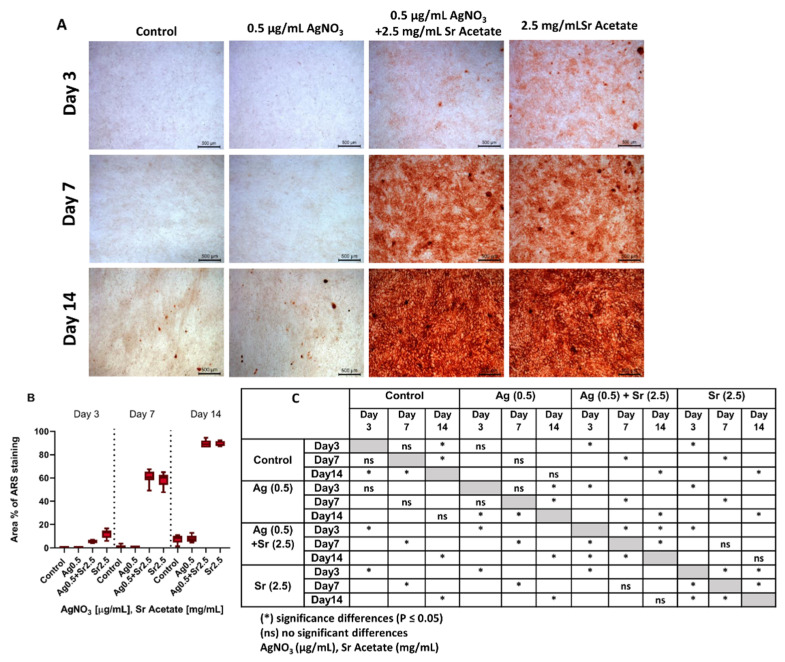
Differentiation of MC3T3 cells. (**A**) Alizarin Red staining results observed by microscopy on day 3, 7 and 14 to show the osteogenesis of MC3T3 treated with AgNO_3_ (0.5 µg/mL), Sr Acetate (2.5 mg/mL) or combination of both. (**B**) Area percentage of MC3T3 cells stained with ARS processed using Image J software. (**C**) Significant differences between groups. Scale bar: 500 µm.

Since MC3T3 cells exhibited morphological changes upon treatment with SrAc alone as well as in combination with AgNO_3_, potential osteogenic differentiation was analyzed both in mono- and co-culture using Alizarin Red staining (ARS). MC3T3 were cultured in an osteogenic medium for up to 14 days and treated with the lowest identified therapeutic combination of AgNO_3_ (0.5 µg/mL)/SrAc (2.5 mg/mL) as well as AgNO_3_ (0.5 µg/mL) and SrAc (2.5 mg/mL) alone. Isolated calcium deposits stained with ARS, which visualized mineralized nodules, appeared in the control and 0.5 µg/mL AgNO_3_-treated cultures only on day 14, whereas days 3 and 7 revealed no mineralization (Figure 5A). There were also no further differences in cell appearance between these two groups, which confirmed once more the cytocompatibility of 0.5 µg/mL AgNO_3_ (Figure 5C). When treated with 2.5 mg/mL SrAc (alone or in combination with 0.5 µg/mL AgNO_3_), at day 3 extracellular matrix mineralization was statistically significantly elevated already in comparison to the control or cultures only challenged with AgNO_3_. However, SrAc alone induced mineralization faster than combined treatment with AgNO_3_ and SrAc, which was apparent through more intense staining on day 3 (Figure 5B). From day 3 onwards, mineralization increased statistically significantly in both groups and reached its maximum after 14 days (approximately 11-fold increase in comparison to cultures without SrAc) (Figure 5C).

## 3. Discussion

Long-term dental implant function depends on both sufficient tissue integration and avoidance of bacterial colonization. Consequently, a great interest has risen in substances with dual functionality, biofilm control as well as cell compatibility. In a recent study, the antimicrobial potential of strontium hydroxide against pathogenic oral bacteria was demonstrated [23], and related reviews reported the antimicrobial effects of functionalized surfaces with silver, strontium, or combinations of both [13,33]. Therefore, the aim of the present study was to systematically describe a therapeutic window for the application of silver and strontium salts on tissue cells and pathogenic bacteria of the oral cavity for the first time.

For this purpose, the antibacterial properties of AgNO_3_ on *A. actinomycetemcomitans* as well as the cytotoxic effects for osteoblasts (MC3T3) and fibroblasts (NIH3T3) were investigated first. Cytotoxicity was quantitatively and qualitatively assessed by measuring metabolic activity and observing cell morphologies using cell staining with CLSM. The combination of both methods provides more information regarding the cytocompatibility of tested chemical substances than analyzing only one parameter. Since bacteria and cell culture media in general are differently composed and the influence of media composition on the toxicity of silver ions was documented in several studies [34,35], this should be taken into account if a putative therapeutic window is to be derived from in vitro data. In the present study, the same culture medium (DMEM + αMEM, 1:1 ratio) was found appropriate for the cultivation of bacteria, as well as different tissue cells, and facilitated the direct comparison of mono-cultures, co-cultures and the effect of different additives. Since the therapeutic ranges of silver in solution were shown to be much smaller than silver derived from coatings [36], it illustrates the importance of using dissolved silver ions for the analysis of mutual functionality. In line with previous studies, results of separate mono-culture experiments showed a linear reduction in the viability of both bacteria and tissue cells reflecting the concentration-dependent toxic activity of silver [37]. The underlying mechanisms include the production of reactive oxygen species (ROS) resulting in cellular disruption and subsequent cell death [6]. From the results presented here for the effect of silver ions in monocultures, only a therapeutic range for MC3T3 could be derived based on the lowest inhibitory concentration for bacteria (MIC) and the highest dose that is still tolerated by cells (Table 1). In contrast, no therapeutic range could be defined when considering NIH3T3 cells. In the context of an intended therapeutic application, it is therefore of great importance to consider all affected tissues as early as possible in the investigations, since various cell types present different sensitivities to silver. In particular, the minimum AgNO_3_ concentration showing cytotoxicity towards MC3T3 cells were found to be 4 times higher than the cytotoxic ones in NIH3T3 cells. Similar observations regarding NIH3T3 sensitivity were reported previously by Sambale et al. [38]. The finding that treatment with AgNO_3_ reveals a therapeutic window in the case of osteoblasts, on the other hand, is much more controversially discussed in scientific journals [37,39,40,41,42,43]. For instance, Kirmanidou et al. showed antibacterial activity against *Porphyromonas gingivalis* and *Prevotella intermedia*, and cytocompatibility for SaOS-2 osteoblast-like cell lines using silver nanoparticle doped titanium surfaces [43]. In contrast, some studies indicated that silver concentrations of higher than cytocompatible doses are needed to observe their bactericidal effects [10,42]. In most cases, this contradiction is probably due to the different study designs, with different bacterial strains, cell lines, media and types of silver administration, which makes cross-study comparability considerably more difficult [38,44,45]. Thus, a therapeutic window always needs to be identified for specific application purposes.

In the same mono-culture setups, the cytocompatibility and antibacterial potential of strontium acetate were also tested. Despite the beneficial osteogenic effects of strontium, which have been extensively investigated, only a few studies evaluated the bactericidal effect of strontium against pathogenic oral bacteria [23,33]. In the present study, a minimum inhibitory concentration of SrAc (5 mg/mL) against *A. actinomycetemcomitans* after 24 h of incubation was defined. These results are in line with findings of Alshammari et al., who reported similar antibacterial results after using strontium hydroxide against six periodontal pathogens [23]. Concentrations in a range of 1.21 mg/mL to 12.1 mg/mL showed complete growth inhibition in planktonic cultures and biofilm assays, respectively [23]. The differences between the bactericidal concentrations in the above mentioned study and the research presented here can be attributed to different experimental designs and chemical forms of strontium. Although the exact process regarding the antibacterial behavior of strontium is not yet fully understood, two possible mechanisms have been proposed. The first mechanism occurs directly through the induction of oxidative stress (ROS) or interruption in adenosine triphosphate (ATP) synthesis, causing DNA damage and ultimately the death of bacteria; the second mechanism works indirectly by changing the physicochemical conditions of the pericellular microenvironment, such as higher osmotic forces and pH change, which are unfavorable for some bacterial strains [23,46]. The specific mechanism responsible for the antibacterial effect observed in this study should be addressed in follow-up studies.

According to the CTB and cell morphology analyses, low doses of SrAc (up to 3.5 mg/mL) can be considered cytocompatible for both investigated cell lines. However, similar to AgNO_3_, the minimum concentration of SrAc which causes more than a 30% reduction in metabolic activity differs between fibroblasts and osteoblasts, revealing the higher sensitivity of NIH3T3. In contrast to silver, the administration of SrAc directly below the cytotoxic concentration (1.5 to 3.5 mg/mL) even leads to a significant increase in the metabolic activity of MC3T3. This observation can probably be attributed to the specific effect of strontium on osteoblasts expressing Ca-sensing receptors (CaSR). Strontium is known to bind CaSR, thereby activating various signaling pathways which are essential in cell proliferation, differentiation, and osteogenic gene expression impacting metabolic activity change [21]. In the present study, a therapeutic concentration (5 mg/mL) of SrAc could be defined, which exhibited antibacterial activity without compromising the cytocompatibility of MC3T3, if not stimulating cell activity. Unfortunately, no therapeutic window could be determined in the case of NIH3T3. Promoted osteogenic responses in MC3T3 cells and bactericidal effects toward *A. actinomycetemcomitans* and *P. gingivalis* have also been reported in a study using Sr-substituted bioactive glasses, even though a synergistic effect of the bioactive glasses could not be excluded [47]. However, at high concentrations the toxic effects of SrAc were observed for both cell lines as well. Accordingly, some studies demonstrated the inhibitive effect of high doses of strontium on cell proliferation and differentiation in an in vitro setup, and interference of high doses of strontium (>510 mg/kg/ day) with Ca metabolism causing hypocalcemia and bone mineralization defects in vivo [21]. In this study, only low doses of strontium in a cytocompatible range were considered, which are also suitable for future investigations regarding clinically safe local application.

After defining therapeutic windows of AgNO_3_ and SrAc in the mono-culture experiments (Table 1), dilutions of AgNO_3_ were combined with an SrAc concentration that can be considered cytocompatible for MC3T3 as well as NIH3T3 (2.5 mg/mL) in order to achieve intensified antibacterial and optimal cytocompatibility results. The concentration of AgNO_3_ in combination with 2.5 mg/mL SrAc, which could inhibit > 99% of bacterial growth in a co-culture setup for both cell lines, was determined to be 0.5 µg/mL. This concentration is six times lower than required to exert the same effect without adding SrAc. As 0.5 µg/mL AgNO_3_ or 2.5 mg/mL SrAc alone were not considered bactericidal in mono-culture experiments, a synergistic antibacterial effect was inferred for combined treatment. Similar synergistic responses were achieved in other studies after the addition of different bactericidal agents to silver, such as antibiotics, bactericidal metals, hydrogen peroxide, etc. [29,48,49,50]. To our knowledge, the exact mechanism behind the synergistic bactericidal effect of silver/strontium combinations still needs to be addressed. This might be due to different antibacterial modes of action. The co-culture system also influenced the antibacterial effect of AgNO_3_ without SrAc. However, a lower effectiveness in the co-culture setup was observed than in the mono-culture experiments. As the effect of different media can be excluded, this finding was probably related to the influence of cells or the co-culture design. It is known that osteoblast lineage can internalize some bacteria and protect them from antibacterial substances [51]. However, no studies have investigated this effect on *A. actinomycetemcomitans*. Additionally, higher total cell numbers (prokaryotes plus eukaryotes) were exposed to the same concentration of chemical substances in the co-culture setup. This might have reduced the available amount of silver for every separate biological unit. On the other hand, the combination of a low concentration of AgNO_3_ with 2.5 mg/mL SrAc also exhibited less cytotoxicity in NIH3T3 and MC3T3 cells compared to AgNO_3_ treatment without SrAc. This phenomenon was also described previously [19,29] and was attributed to the ability of strontium to occupy specific binding sites prior to silver which can directly influence cellular functions or indirectly activate cell proliferation signaling pathways [52]. In contrast, high concentrated AgNO_3_ in combined treatment with SrAc led to a significant decrease in cytocompatibility, which might represent a turning point in the mitigating effect of additive SrAc, which was observed for both cell lines albeit in different concentration ranges due to varying sensitivity levels. The ambivalent effect of SrAc on cells and the synergistic antibacterial effect of combined treatment (AgNO_3_/SrAc) might raise concerns regarding the clinical application where not only several factors can change the concentration of these two chemicals, subsequently missing the therapeutic range, but also adverse effects such as allergic reactions might occur due to their combination. So far, no other study has been reported that compared various cell responses and their susceptibility following the use of AgNO_3_/SrAc with different concentrations. However, some investigations were carried out reporting varied silver sensitivities in mammalian cell lines and improved cytocompatibility in fibroblasts and osteoblasts after using low doses of Sr [21,38,53]. This could be due to different amounts of ROS formation, but a detailed analysis would be required for further investigation.

As in all experimental setups, morphology alterations in MC3T3 after SrAc treatment and increased metabolic activity in combined treatment with low concentrations could be detected. Their potential effect on osteogenic differentiation was analyzed by matrix mineralization staining. Indeed, clearly increased mineralization rates could be observed in cultures containing SrAc at all time points. This is in agreement with previous studies, which already reported on the osteogenic potential of strontium [21]. The slightly delayed mineralization for combined treatment might be related to the time-dependent impact of AgNO_3_. Other studies showed lower cell viability and proliferation during the initial days of treatment due to the differential uptake of silver into the cells [54,55]. In contrast, no significant differences in osteogenic differentiation could be detected between the untreated control and AgNO_3_ stimulus alone which is in line with the literature for low concentrations [56].

The subsequent therapeutic windows of an AgNO_3_ treatment in combination with SrAc (2.5 mg/mL) shifted in the case of MC3T3 to 0.5–3 µg/mL, while for NIH3T3 it was described for the first time (0.5–1.5 µg/mL). Considering hard and soft tissue in an oral application, 0.5–1.5 µg/mL of AgNO_3_ together with 2.5 mg/mL SrAc can be considered as an expedient treatment. Notably, no therapeutic range (for both AgNO_3_ and SrAc) was detected in NIH3T3 in separate treatments, nor were any of these concentrations initially defined as therapeutic for both cell lines in mono-culture experiments (Table 1). Taken together, beside a synergistic antibacterial effect and increased therapeutic window, combining AgNO_3_ with SrAc also seemed to stimulate the differentiation of osteogenic cells. When comparing the results of this study—the synergistic dual function of AgNO_3_/SrAc with additional osteogenic effect—with the literature, it is apparent that different studies already used silver/strontium combinations to benefit from the antibacterial characteristics of silver and bone formative properties of strontium [20,24,27]. However, there are some differences between the current study and previously reported strategies. First of all, most strategies involved surface coatings instead of an analysis of soluble substances which does not allow for an accurate conclusion on the effect of these chemicals due to the background effect of the coating. Secondly, none of these studies reported antibacterial effects following the use of strontium alone. Moreover, controversial results of the synergistic antibacterial activities and enhanced therapeutic windows of silver/strontium combinations were reported [20,28,29]. In this regard, Van Hengel et al. observed an osteogenic response and synergistic antibacterial action against methicillin-resistant *Staphylococcus aureus* (MRSA) on a porous implant surface biofunctionalized with SrAc and silver nanoparticles while no significant difference in osteogenic activity of porous implant surfaces with and without strontium was observed [29]. However, in a different study, the substitution of silver and strontium in hydroxyapatite (HA) coatings showed no synergistic antimicrobial properties [28]. This emphasizes the impact of the coating strategy itself on the expected antibacterial and osteoinductive effectiveness. Therefore, this needs to be taken into account if desirable combinations of AgNO_3_ and SrAc as defined in the present study are going to be applied in an innovative implant surface strategy. The established systematic analysis of synergistic combinations of active ingredients will guide this process. It also sets the basis for a variety of studies that will address the exact molecular mechanisms behind the cytotoxicity and antibacterial effects of silver and strontium, which remain unknown to this date. The aspect of more complex bacterial compositions, such as biofilms instead of planktonic bacteria, and different interactions between cellular and extracellular environments in tissue in comparison to monolayer cell cultures is considered as another area requiring investigation on the way to successful clinical application.

## 4. Materials and Methods

### 4.1. Preparation of Silver Nitrate and Strontium Acetate Solutions

Silver Nitrate (AgNO_3_, BioXtra, >99%) and Strontium Acetate (SrAc, Sr(CH_3_CO_2_)_2_) were obtained from Sigma-Aldrich^®^ (Merck KGaA, Darmstadt, Germany). Solutions were freshly prepared by dissolving them in sterile Milli-Q water and further diluted to the final concentrations (Table 2) in experimental volume (vol/vol not exceeding 2.5%).

### 4.2. Determination of Cell Viability and Morphology in Mono-Culture

Immortalized murine fibroblasts (NIH3T3) and immortalized murine pre-osteoblasts (MC3T3-E1) obtained from American Type Culture Collection (ATCC, Manassas, VA, USA) were used in this study. Both cell lines were cultivated in a mixture of Dulbecco’s modified Eagle’s medium (DMEM, Cat. No P04-04500, PAN-Biotech, Aidenbach, Germany) and Alpha Minimum Essential Medium (αMEM, Cat. N° BE02-002F, Lonza, Cologne, Germany) (in a 1:1 ratio) supplemented with 10% fetal bovine serum (FBS, PAN-Biotech GmbH,) (*v/v*) and 1% penicillin/streptomycin (P/S, Sigma-Aldrich, Steinheim, Germany) under a humidified environment at 37 °C with 5% CO_2_. The cells at passage numbers 7–13 were allowed to grow to up to 80% confluence, trypsinized and adjusted to 1 × 10^5^ cells/mL before being seeded in experiments. All cell experiments were performed in three independent biological replicates. Cells cultured without any test substances served as controls.

#### 4.2.1. Cell Titer-Blue (CTB) Cell Viability Assay

The viability of tissue cells was assessed using a CTB assay kit (Promega, Madison, WI, USA). Cells were seeded in 96-well microtiter plates (CELLSTAR-Greiner Bio-One, Kremsmünster, Austria) with a density of 1 × 10^5^ cells/mL (100 µL/well). They were incubated for 24 h at 37 °C in 5% CO_2_. The medium was changed the next day and cells were treated with different concentrations of AgNO_3_, SrAc, or combinations of both which had been previously dissolved in medium. They were incubated for another 24 h under the same conditions. A CTB-assay was performed based on the manufacturer’s protocol. Briefly, 20 µL of CTB reagent was added to seeded cells and the plates were incubated at 37 °C for 4 h. The metabolic reduction of resazurin to fluorescent resorufin was recorded at 560/590 nm using a plate reader (Tecan, Infinite M200Pro, Männedof, Switzerland).

#### 4.2.2. Cell Morphology Analyses by Confocal Laser Scanning Microscopy

Live/dead staining of both cell lines was performed to confirm cell compatibility and evaluate cell appearance/morphological change following exposure to test substances. Cells were seeded in 6-well plates (CELLSTAR-Greiner Bio-One, Kremsmünster, Austria) with a density of 1 × 10^5^ cells/mL (3 mL/well) and incubated at 37 °C in 5% CO_2_. The medium was changed the next day and cells were treated with different concentrations of AgNO_3_, SrAc, or combinations of both for 24 h. Finally, cells were fluorescently stained using SYTO9 and Propidium Iodide (PI) (BacLight Bacterial Viability Kit, Thermo Fisher Scientific GmbH, Dreideich, Germany). The working staining solution was prepared in 1:1000 dilution by adding SYTO 9 and PI into phosphate-buffered saline (PBS, Sigma-Aldrich^®^). Stained cells were fixed using 2.5% glutaraldehyde (Roth, Germany; 1:10 dilution with PBS) for 30 min at 4  °C. Images were taken by performing confocal laser scanning microscopy (CLSM; Leica TCS SP8, Leica Microsystems, Mannheim, Germany).

### 4.3. Determination of Antibacterial Activity in Mono-Culture

*Aggregatibacter actinomycetemcomitans* (ATCC 33384) was obtained from the American Type Culture Collection (Manassas, USA) and routinely stored as glycerol stocks. Bacteria were cultured for 48 h on fastidious anaerobe agar (FAA, Oxoid Limited, Wesel, Germany) plates with 5% sheep blood (Thermo Fisher Scientific, Germany) under anaerobic conditions. Colonies from FAA plates were inoculated into brain heart infusion medium (BHI; Oxoid Limited) supplemented with 10 μg/mL vitamin K (Carl Roth GmbH & Co. KG, Karlsruhe, Germany) within an anaerobic chamber (80% N_2_, 10% H_2_, 10% CO_2_ and 37 °C). The overnight pre-culture was centrifuged and resuspended in phosphate-buffered saline (PBS, Sigma-Aldrich^®^) to a final optical density (OD) of 0.1 at 600 nm. A total of 15 µL of bacterial suspension was mixed with 3 mL mixed cell culture medium (DMEM + αMEM, 1:1) containing 10% FBS (*v/v*) without antibiotics (P/S) and seeded in 96-well microtiter plates. AgNO_3_ and SrAc were added in different concentrations and combinations (Table 2) to evaluate the minimum concentration able to inhibit bacteria growth. After 24 h of incubation at 37 °C and 5% CO_2_, serial 10-fold dilutions in PBS were made from each well. Aliquots of 100 µL of each dilution were plated on FAA plates (+ 5% blood) and incubated at 37 °C and 5% CO_2_ for 48 h. The colony-forming units (CFU/mL) were counted from the plates. As a form of growth control, the bacteria were cultivated without Ag and Sr additives. Three independent experiments each with an intra-experiment duplicate were carried out.

### 4.4. Co-Culture of NIH3T3/ MC3T3 with A. actinomycetemcomitans

NIH3T3 and MC3T3 were cultured separately in 6-well plates with a concentration of 1 × 10^5^ cell/mL (3 mL/well) as described previously. *A. actinomycetemcomitans* was inoculated in BHI broth supplemented with 10 μg/mL vitamin K under anaerobic conditions (80% N_2_, 10% H_2_, 10% CO_2_ and 37 °C) on the same day. After 24 h, the bacterial suspension was centrifuged, resuspended in PBS and adjusted to the desired optical density of 0.1 measured at 600 nm. A total of 15 µL of this suspension was used to challenge both cell lines representing a multiplicity of infection (MOI) of 5:1 (bacteria:cells). Different concentrations of AgNO_3_, SrAc, or a combination of both were added to the wells, while the control groups received a medium without test substances. The co-cultured cells were incubated for 24 h at 37 °C and 5% CO_2_. Subsequently, CFU counting and cell staining were performed as described above for mono-culture experiments. The experiments were carried out in three independent repetitions.

### 4.5. Characterization of Cell Differentiation

Osteogenesis was characterized by the extracellular calcium deposition of MC3T3-E1 cells (Alizarin red S staining, ARS). MC3T3-E1 cells were seeded in 24-well plates (CELLSTAR-Greiner Bio-One) with a density of 1 × 10^5^ cells/mL (1 mL/well) and grown to >100 % confluence. Subsequently, the medium was replaced with an osteogenic differentiation medium consisting of the basic culture medium supplemented with 10% FCS, 1% P/S, 0.1 mM Ascorbate (Sigma-Aldrich^®^, Merck KGaA), 5 mM β-Glycerophosphat (Sigma-Aldrich^®^, Merck KGaA), 1.8 mM KH_2_PO_4_ (Carl Roth GmbH & Co. KG), 10 nM Dexamethason (Sigma-Aldrich^®^, Merck KGaA) and the respective AgNO_3_ and SrAc concentrations as specified in the results section every 3 days. After 3, 7, and 14 days, cells were washed three times with PBS and fixed with a 4% paraformaldehyde solution (Carl Roth GmbH & Co. KG) for 20 min at room temperature. After washing thrice with diH_2_O, the cells were stained with 2% Alizarin Red S (pH 4.2) (Sigma-Aldrich^®^, Merck KgaA) for 30 min at room temperature. The cells were washed an additional four times with diH_2_O and finally covered with 200 µL PBS. The qualitative examination was performed by imaging stained cells under an optical microscope (Leica DMi1) using 10-fold magnification objective and LAS V4.8 software. Images were quantitatively analyzed using Image J software (National Institutes of Health, Bethesda, MD, USA). The blue channel, which corresponds to ARS staining, was used to threshold images and the stained area percentage was processed subsequently. Three individual experiments each in quadruplicate were performed.

### 4.6. Statistical Analysis

GraphPad Prism Software 8.4 (GraphPad Software Inc., La Jolla, USA) was used to perform statistical analysis and graphic processing on the data. All data were checked for normal distribution using the Kolmogorov–Smirnov test. For all mono-culture and co-culture experiments, the significant differences with the control groups were determined by conducting a Kruskal–Wallis test. The Mann–Whitney U test was used to define the significance between AgNO_3_ and AgNO_3_/SrAc treated groups in a co-culture setup. A two-way analysis of variance (ANOVA) was used to compare groups over all time points in the ARS experiment. The significance level was set to α = 0.05 for all comparisons.

## 5. Conclusions

The results presented here on the antibacterial and cytocompatible properties of AgNO_3_ and SrAc clearly defined (i) an optimum AgNO_3_ concentration that can impede the growth of bacteria associated with peri-implantitis while exhibiting cell compatibility, (ii) an optimum SrAc concentration exerting osseoinductive and antibacterial effects, and (iii) an enhanced therapeutic window of AgNO_3_/SrAc combination. Herein, using AgNO_3_ and SrAc with low concentrations resulted in cytocompatible properties with potential antimicrobial activity. Interestingly, by combining non-therapeutic concentrations of these two chemicals (AgNO_3_, SrAc), we observed a synergistically intensified antibacterial effect, cell compatibility, and osteogenic differentiation in a co-culture model. The results also suggest that silver/strontium coating of implants may be a promising therapeutic strategy for the prevention of peri-implantitis and poor osseointegration and therefore requires further research.

## Figures and Tables

**Table 1:** Therapeutic windows of Silver Nitrate, Strontium Acetate and their combination.

	Chemical	AgNO_3_ (µg/mL)	SrAc (mg/mL)	AgNO_3_/SrAc * (µg/mL)
Cell Line				
NIH3T3		---	---	0.5–1.5
MC3T3		1.5–3	5	0.5–3

* Combined treatment with AgNO_3_ in presence of SrAc 2.5 mg/mL.

**Table 2:** Chemicals concentrations in all experimental setups.

	Setups	Mono-Culture	Co-Culture
Chemical			
AgNO_3_ (µg/mL)		0.5, 1.5, 3, 6, 9, 12, 15, 20	0.5, 1.5, 3, 6
SrAc (mg/mL)		0.1, 0.5, 1, 1.5, 2.5, 3.5, 5, 20	2.5

## Data Availability

Data are available upon reasonable request from the corresponding authors.
